# Eugenic Parenthood and Its Relation to Christian and Social Ethics

**Published:** 1914-03

**Authors:** 


It gives me great pleasure to present to my readers this month what I consider one of the best papers ever written on "Eugenic Parenthood and Its Relation to Christian and Social Ethics." As I have read and reread the paper I have had visions of a regenerated race out of whom has been bred the predisposition to evil. It is a glorious gospel that this brave little woman is bringing us, and I wish it were in my power to place it in the hands of every intelligent woman in the United

States. I ask that you will read it carefully and then lend it to your neighbors and friends. Put it away and read it to your sons and daughters before their minds become contaminated by evil associations. Read it to your husbands. Of course they will ridicule the idea at first, but by patience and firmness and by enlightening your own mind on these subjects you may be able to convince them that Mrs. Teats has pointed the way by which you may give to the world nobler and better sons and daughters.

No man or woman can read this article thoughtfully without catching a glimpse of procreation as it should be, and man as the offspring of such parenthood reflecting more than ever before the divine image of the Creator.

JOSEPHINE DANIEL.

---

Written exclusively for the Texas Medical Journal.

### Eugenic Parenthood and Its Relation to Christian and Social Ethics.

In the discussion of this subject I have taken the liberty to quote largely from up-to-date authorities, upon the subject of "Intelligent Parenthood," and also from the fuller interpretation of God's word.

### MARRIAGE.

"Marriage is co-partnership in social service. It should be regarded as a means of realizing the highest spiritual life, a life-long companionship of love and labor, in which the rearing of children is the greatest privilege."

### WHAT IS PARENTHOOD?

"A partnership with God is parenthood;
What love, what purity, what self-control;
What wisdom, what knowledge should belong to those
Who help God fashion an immortal soul!"

In the above quotation we find five essential characteristics for intelligent parenthood. First is love. "Love worketh no ill to his neighbor." A man can have no neighbors closer than wife and children.

Professor Newton N. Riddell gives the following definition of love: "Pure love as it proceeds from the spirit is holy, both in character and expression. It is vitalizing, strengthening, recuperative and constructive in every faculty and function. It is the fountain of life, springing forth from the divine in man; the source of all the higher virtues, which in their sum total form the image of God. Love perverted chemicalizes in lust, which is destructive, degenerative and devitalizing. The perverted, or poisoned, fountain is the course of bitterness, pride, envy, jealousy and all the evil tendencies which in their sum total form the image of Satan in man. Satan, by perverting this fountain of life, perpetuates degeneracy through the generative functions, and by constantly poisoning this fountain makes vice natural and virtue difficult. Through sexual degeneracy he continually begets his own, and brings forth his image and likeness in the Son of Man, who should be the image and likeness of God."

Havelock Ellis says: "Whenever men and women stand in each other's presence the sexual instinct will always insure an adequate ideal halo." In other words, if we generate purity and love within, we will radiate purity and love without, and all with whom we come in contact will feel its influence.

In harmony with the foregoing, the vast differences between sexuality and sensuality should be thoroughly understood. Sexuality is of God and is as sacred and sublime as its author. Sensuality is of the adversary, and is as hideous and destructive as its author. Sexuality is love lawfully focussed, and is God's dynamo for the production of the "more abundant life." Sensuality is sexuality unlawfully focussed and produces the "more abundant death," for nowhere in God's economy is waste any part of natural life.

"Be not deceived, God is not mocked, for whatsoever a man soweth, that shall he also reap." If he soweth to the flesh (sensuality), he shall of the flesh reap corruption (sensuality). "But he that soweth to the spirit (love) shall of the spirit reap life everlasting."

Second, purity. "To the pure all things are pure," including the powers of parenthood, a gift of God. Parenthood must become a vocation, and both love and parenthood must become intellectualized and scientific. The science of life is God's law; and sin is the transgression of law. Let us preserve within us the image of the divine and of public health, the two guardian angels of prevention from without, and vitality from within. It therefore behooves us to guard well this inner force that protects the citadel of our lives.

Third, self-control. The self-control "can do all things through Christ which strengtheneth him," including control of the powers of parenthood.

Fourth, knowledge. "Get knowledge" is the command, especially knowledge concerning God's laws of life and reproduction.

Fifth, wisdom. "With all thy getting, get wisdom." There is no vocation in life where wisdom is more sadly needed than by those who contemplate the establishing of a home, and furnishing a citizenship for two worlds, for time and eternity.

Frances E. Willard said: "I believe in the divinity and dignity of sex, and in the free and full discussion of all that pertains to the nature of man, or is essential for his well being." The same brave soul also claimed that "That most holy thing in all the world, the wedded love of two, is murdered by the deadliest lust."

A judge of the Domestic Relations Court of Chicago, in a public address which I was privileged to hear, corroborates Miss Willard's statement by saying that "The primary cause of disharmony in the home, resulting in divorce, is the untellable abuse in the marriage relation to which I have to listen, and which cannot be printed. Hence the world does not know the real cause of a majority of divorces."

G. Campbell Morgan says: "Animalism has been for ages the curse of the marriage relation." Quoting from the eugenic department of "The Homiletic Review," said department being edited by Dr. Josiah Strong, we find the following statement: "From time immemorial, the question of the true relation of the sexes has not only been relegated to the realms of mystery, but covered with a sevenfold veil of shame. This sense of shame is born of a consciousness of guilt. One sees this forcibly illustrated in the story of the first sin, and its results, as recorded in the book of Genesis."

At this point may not the question be reasonably asked what are we to conclude was the basic cause of man's fall. Disobedience? Yes. But disobedience to what? It it reasonable to suppose that God ever settled the eternal destiny of a human race on so insignificant a test of obedience as eating any kind of fruit, of God's own creation? Is it not reasonable to conclude that Satan in his effort to bring forth death and the destruction of the race would naturally and logically strike the blow at the fountain of life?

To the extent that our first parents failed to conserve God's elixir of life for the God ordained purposes: First, to build up the individual to his highest possible attainment and efficiency; and, second, to comply with God's command to "multiply and respenish the earth and subdue it"; just to that extent did Adam and Eve die, and the tragedy has continued all down the ages, to a greater or less degree. Surely, the vast army of degenerates and the unemployable are rather poor material from which to "multiply" and "subdue the world."

From reliable sources we have the statement that degeneracy is increasing two and one-half times faster than the population is increasing. Let us hope that these figures are exaggerated. But even if half true, does it not behoove us to see to it that we set a higher standard for parenthood? We must cleanse the fountain, the home, if we would have the stream, social, governmental and religious life, pure.

Adam and Eve "set the pace that kills," physically, mentally and spiritually, through the dissipation of God's elixir of life. Christianity must set the pace that will kill the social evil, as our first parents set the pace that started the social evil.

The full extent and the holier meaning of the sacrifices God made to redeem man from the sin of violated sexual law, seems to have been in the past, the most difficult problem for man to solve. Even Paul cries out (Romans 8:22, 23): "For we know that the whole creation groaneth and travaileth in pain until now; and not only they, but we ourselves also (i. e., Christians), which have the first fruits of the spirit, even we ourselves groan within ourselves, waiting for the adoption, towit, the redemption of our bodies." Herein lies the tragedy of Christian civilization, namely, that with some noble exceptions children have been begotten in Christian homes on the same plane as in non-Christian homes. True, the children of Christian parents have had the blessed advantage of Christian training, before and after birth, and it is to this fact that we owe our high standard of Christian civilization. I am sure that the average parents of the past, as well as of the present, aim to do the best they know for their children. So no one is justified in passing harsh or unkind criticisms, for have we not all sinned (on this line) and come short of the glory of God, in so marring and scarring His image in Man? But our ignorance of nature's laws does not prevent our suffering the full penalty of violating her laws.

These divine laws were understood and taught by our early Christian fathers, who came in close touch with the teaching of the gentle Christ and the apostles; and these principles were embodied in the apostolic constitution, as follows: "And incontinence is the destruction of one's own flesh (an up-to-date scientific fact), not being made use of for the procreation of children, but for pleasure, which is a mark of incontinency, and not a sign of virtue. The conjunction of the sexes is agreeable to God for the sake of procreation alone."

If the foregoing had been the rule of Christian parenthood from the time these brave words were uttered, I am going to say without fear of successful contradiction that we would today be such physical, mental and spiritual giants that all the powers of his satanic majesty could not stop the onward march of the Church of Jesus Christ!

How can First Corinthians, 7:14, be interpreted otherwise than that a parenthood wholly sanctified, both body and soul, should give to children Godward tendencies? J. Wilbur Chapman, the world-renowned evangelist, stated some years ago through the religious press, that "Christians ought to have been born centuries ago."

Dr. John T. McFarland, in "The Book and the Child," asks this important question, "May not the child's spiritual life be made continuous as well as his physical and mental life?" And makes the statement that there is an abundance of evidence to prove that such can be the case. If this be true, then where does the responsibility rest? In short, he claims that Christians, from a sanctified parentage, ought to be born, but states that the time must come when children so favorably born, must take a stand before the world and "acknowledge Christ as their master."

"A young man before the ordaining council was asked the usual question, 'Can you state the time when you were converted and came into a state of grace?' He answered, 'No, for I was born of a God-loving and a God-obeying parentage, and so I was born in a state of grace.'" It is said that the answer was so sincere and truthful, and opened up a vista of such glorious possibilities of a larger doctrine than the questioners held, that it was allowed to pass.

Permit me to give, in the form of an equation, what that larger doctrine may be, namely, regeneration, plus right generation, will equal human redemption. Who can measure the glorious possibilities vested in man when he is thoroughly "in tune with the Infinite." "All things are possible when God and man join together to do the impossible."

Drummond says: "The Christian, like the poet, is born, not made."

Dr. C. F. Hodge, professor of biology of Clark University, in a very able discussion of the sex problem, says: "So here we have the ultimate ground for unlimited, careless, easy, unwholesome marriage, with its corresponding relief in divorce; and as the running sore of this abnormal condition, we have promiscuity and prostitution. The latter are but the incidental ills, from a bad philosophy of sex, and do not tend to thwart and corrupt the great stream of social evolution as do anti-biological practices in the marriage relation itself. We need sublimation of sex quite as much after marriage as before it." "Perfect power of control before marriage means power of control after marriage." "Moral will of yesterday is moral instinct of today."

As a race progresses in civilization, its children are born at a higher intellectual and moral average. "The law of life is progress. The law of degeneration is ultimate elimination." Granting that such a thing were possible, it would be a Godsend to humanity if not another child were to be born in five years, our forces to be centered upon properly caring for the millions of children already in existence and educating our young people for intelligent parenthood. The following statement was made before a large audience by Prof. Dallas L. Sharp of Boston University: "Children nowadays, though as welcome as ever when they ho happen to appear, are incarnate accidents."

Chief Justice Harry Olson of Chicago, says: "If the slums were done away with, and fine apartments erected in their place, the slums would return, because it is not a question of poverty but of blood." And we may add that bad blood is largely the result of ignorant parenthood.

Emerson says: "The soul's economy is to spend for power, not for pleasure. The body is a vessel in which the elixir of life is stored. Imperious necessity is a monster delusion. Not human or sensual needs, but human rights, should be the guide in reproduction."

I have in my possession the names of seventy-five physicians of New York City, signed to the following statement: "In view of the widespread suffering, physical disease and moral deterioration inseparable from unchaste living, the undersigned members of the medical fraternity of New York and vicinity unite in declaring it as our opinion that continence, and a pure, clean life for both sexes, is consonant with the best conditions of mental, physical and moral health."

So long as children are the product of selfishness, just so long we will have a selfish humanity, resulting in greed, get, gain and graft. When children are the product of generosity instead of selfishness, giving of our life that another soul may be, then the Golden Rule of Christ will take the place of the rule of gold!

A subjected motherhood can never give to the world the best results in child life. While filling an engagement of fifty-one lectures in New York City, I spent much time in investigating social conditions, especially on Blackwell's Island, where, on one occasion, I saw 2100 incorrigible prison boys, under heavy guard, taking open air exercises. These boys were all in their teens. Surely these 2100 incorrigible boys could not have been the product of intelligent parenthood. Is it not reasonable to suppose that they were the offspring of a rebellious motherhood, and that rebellious motherhood is the result of a subjected wifehood, and that subjected wifehood is the legitimate result of the monstrous and outrageous physical necessity and marital rights theories for man? Theories that must have been conceived in an extra session of his satanic majesty's congress, for I doubt if Satan himself could have invented theories so disastrous to the human race, entirely unaided! God never created man with "necessities" that involved the depletion of his own efficiency, the degradation of wifehood and motherhood, and the robbing of a soul of its right to be well born. As Emerson says: "To a well born child all the virtues are natural and not painfully acquired."

It would seem that the impossibles have been accomplished by man in the past half century. He has practically "made the whole world akin" by steam and electricity; he flashes messages from continent to continent under water, and by wireless above water; he tunnels through the mountains; and in a Zeppelin airship, by having discovered the air currents, scales the skies, traveling 200 miles in 117 minutes, piercing the sky a distance of two miles; he has been able to remove man's stomach and heart, replacing them and putting him on his feet again; all these and other innumerable and equally great problems have been solved by the marvelous and mighty "brain and brawn" of man.

But the problem of how to stop the production of the "incorrigibles," the imbeciles and insane, the epileptic and criminal of all types, and degeneracy in general, seems in the past to have been a problem impossible of solution. However, we have every reason to thank God and take courage, for never in the history of the world have there been as many safe, sane and scientific agencies at work for human betterment as at present. The holy sacrament of marriage is being safeguarded as never before. The only way to successfully settle the divorce question and stop the production of the vast army of degenerates, is to settle the marriage question intelligently.

Dean Sumner says: "Half the young men are unfit for marriage." Marriage must be safeguarded by requiring that the applicants for a marriage license possess a reasonable amount of knowledge concerning the divine laws of life and procreation, and that they obtain certificates of good health to the effect that they are free from communicable or inheritable diseases. Also, the segregation or the sterilization of the unfit will be necessary, not as a punitive measure, but in the interest of future generations. It might also be well to apply the Denet test to those securing a marriage license, to ascertain if the applicants, even though of legal age, or beyond, might not be merely children in intellect and efficiency.

I am optimistic enough to believe that, in the not distant future, the ministers of the gospel will be required not only to understand, but to preach God's scientific laws of right generation, in connection with His gracious plan of regeneration; for any theology, or even any system of education, that does not teach and preach God's laws of life and reproduction, must of necessity be a defective theology and a defective system of education.

In Cleveland, Ohio, October 8, 300 members of the Ohio Mothers' Congress pledged themselves not to allow their sons and daughters to marry without medical certificates. They disapprove the slit skirts, and recommend sex hygiene instruction in the public schools.

The Texas Society of Social Hygiene says: "Eugenics is a new religion." The motto of the society is, "The Child Well Born." Their creed, "The exaltation of womanhood and the protection of motherhood by fatherhood; the virtue and virility of woman as the type of the race; the continence of man as necessary to the highest aim of reproduction"; their slogan, "The deliberate and intelligent control of the offspring, the sterilization of the criminal," and many other good things too numerous to mention. Their apology, they say, "We have none."

The latest eugenic movement is the prenatal restaurant recently started in New York City, where prospective mothers can secure proper food in the interest of their unborn children.

Luther Burbank is in hearty accord with the principles and quotations presented in this paper. In a personal interview I had with him in his home, after stating to him my views upon the various phases of the science of life and the eugenic movement, he stated to me in the presence of Dr. Kellogg, professor of science in Leland Stanford University, that the stand I took was absolutely and scientifically correct. To verify this, he gave me a letter of recommendation, from which I quote the following. After favorable preliminary remarks, he says: "Thousands of the most intelligent ones are now following her teachings, and before any great betterment of the human race can be made, her good and true teachings must be generally heeded and followed."

"But a new age looms into sight, like some splendid ship long waited for," the age of genetics, eugenics, and euthenics, embracing the true science of life, the laws of hereditary transmission, ethical marriage, scientific parenthood, prenatal influences; and these, coupled with the higher interpretation of God's word, will result in well born children. This is the slogan of the new age, as well as the new century, upon which we are just entering. Almost innumerable agencies are at work to make this the grandest age under God's direction in human history.

Some churches have engenic classes for their young people. Educational institutions are giving sex instruction in some form. The local eugenics school of Boston, the Correspondence School of Gospel and Scientific Eugenics of Chicago, which, although only four years old, has ever 3000 students from five nations; the Eugenic Record office on Long Island, a department of the American Breeders' Association; the Child Welfare and Little Mothers' Schools; the daily press and magazines, and not least among the helpful features are the new woman and the new man.

The new woman is the woman of the past age, with her great big heart, plus her head. And the new man is the man of past ages, with his great big head, plus his heart; and this combination spells home harmony; and home harmony, in its highest sense, will insure a higher standard of Christian and social ethics founded upon the principles of Christianity and intelligent parenthood contained in the following by Dr. C. F. Hodge: "We must keep the germ plasm on the up grade, free from taint at any cost. This is the supreme law of evolution. To pass on the torch of life not only undimmed, but ever brighter from generation to generation, is the highest service which parents of any generation can render."

While filling an engagement of two months in California under the auspices of the Young People's Christian Societies, I spoke on a Sabbath afternoon at their State convention on "The New Home of the New Century." In closing, I gave the prayer that Dr. Cowan says should be in the hearts of all prospective parents. At the close of the prayer Prof. Joy sang very sweetly:

"More purity give me, more strength to o'ercome,
More freedom from earth stains, more longing for home;
More fit for the kingdom, more used would I be;
More blessed and holy, more, Saviour, like Thee."

After which the benediction was pronounced. A quiet hush seemed to pervade the entire audience, as with bowed heads they listened to the following prayer, after which there was no talking, but people were serious and thoughtful. I trust this may be the spirit of this gathering at the close of the prayer, which is as follows: "O God, our God, this morning, in the full glory of Thy ever-present presence, we supplicate the aid of Thy love, Thy power, in this our soul's desire. Of and from Thee, let the light come that is to guide this child into life, through life, and into eternity. Make it, as we so earnestly desire, a bright, happy, loving, beautiful child. Implant in its organism the qualities that will enable it to live and to grow towards perfection. Give it, we pray Thee, the elements of an holy life, so that all its words, thoughts and actions, will be a reflex of Thy ever-present loving care. And to the end of doing these, our desires, impart, oh God, firmness of purpose, purity of life, and the full measure of Thy daily and hourly presence. And to Thee, Thou Ancient of days, shall be the honor and glory and the praise. Amen."

---

CHICAGO, ILL., February 10, 1914.

*Dr. F. E. Daniel, Editor "The Texas Red Back," Austin, Texas.*

MY DEAR DR. DANIEL: Yours of the 2d inst. was duly received. I am sure we all have cause to rejoice in the rapid strides being made in this almost world-wide movement for more intelligent parenthood and better child life. I have quoted your magazine many times, because I have considered it a matter of vital importance when any of the medical journals take up this advanced educational movement; and you surely are doing your share.

Am sorry that we felt obliged to discontinue our ad in the *Texas White Ribbon,* but it was not bringing us in any returns. I was in hopes that the State where such splendid work is being done on these lines would result in some scholarships for our school. However, they may come later.

Am inclosing you herewith the article of which I spoke in my letter, "Eugenic Parenthood and Its Relation to Christian and Social Ethics." Inclose stamps for its return after you have used it, providing you feel disposed to use it.

Thanking you for the many courtesies we have received at your hand, and wishing you all possible success, I am,

Very sincerely yours,

M. E. TEATS.

